# Strengthening Cellulose Nanopaper via Deep Eutectic Solvent and Ultrasound-Induced Surface Disordering of Nanofibers

**DOI:** 10.3390/polym14010078

**Published:** 2021-12-26

**Authors:** Elizaveta V. Batishcheva, Darya N. Sokolova, Veronika S. Fedotova, Maria P. Sokolova, Alexandra L. Nikolaeva, Alexey Y. Vakulyuk, Christina Y. Shakhbazova, Mauro Carlos Costa Ribeiro, Mikko Karttunen, Michael A. Smirnov

**Affiliations:** 1 Institute of Macromolecular Compounds, Russian Academy of Sciences, V.O. Bolshoi pr. 31, 199004 Saint Petersburg, Russia; batischevaelisaveta@gmail.com (E.V.B.); darya.sokolova.2014@mail.ru (D.N.S.); fedotova.veronicka2016@yandex.ru (V.S.F.); pmarip@mail.ru (M.P.S.); alexandra.l.nikolaeva@gmail.com (A.L.N.); lesha123480@gmail.com (A.Y.V.); hristka11@yandex.ru (C.Y.S.); 2 Institute of Chemistry, Saint Petersburg State University, Universitetsky Pr. 26, Peterhof, 198504 Saint Petersburg, Russia; 3 Departamento de Quìmica Fundamental, Instituto de Quìmica, Universidade de São Paulo, Avenida Professor Lineu Prestes 748, São Paulo 05508-000, Brazil; 4 Department of Chemistry, The University of Western Ontario, 1151 Richmond Street, London, ON N6A 5B7, Canada; 5 Department of Physics and Astronomy, The University of Western Ontario, 1151 Richmond Street, London, ON N6A 5B7, Canada; 6 The Centre of Advanced Materials and Biomaterials Research, The University of Western Ontario, 1151 Richmond Street, London, ON N6A 5B7, Canada

**Keywords:** bacterial cellulose, deep eutectic solvents, cellulose nanofibers, mechanical properties, ethylene glycol, glycerol

## Abstract

The route for the preparation of cellulose nanofiber dispersions from bacterial cellulose using ethylene glycol- or glycerol-based deep eutectic solvents (DES) is demonstrated. Choline chloride was used as a hydrogen bond acceptor and the effect of the combined influence of DES treatment and ultrasound on the thermal and mechanical properties of bacterial cellulose nanofibers (BC-NFs) is demonstrated. It was found that the maximal Young’s modulus (9.2 GPa) is achieved for samples prepared using a combination of ethylene glycol-based DES and ultrasound treatment. Samples prepared with glycerol-based DES combined with ultrasound exhibit the maximal strength (132 MPa). Results on the mechanical properties are discussed based on the structural investigations that were performed using FTIR, Raman, WAXD, SEM and AFM measurements, as well as the determination of the degree of polymerization and the density of BC-NF packing during drying with the formation of paper. We propose that the disordering of the BC-NF surface structure along with the preservation of high crystallinity bulk are the key factors leading to the improved mechanical and thermal characteristics of prepared BC-NF-based papers.

## 1. Introduction

Environmental pollution and the excessive depletion of fossil fuel resources [1] have generated increased interest in renewable and sustainable polymers [2,3]. Cellulose has been regarded as a potential polymer for replacing non-renewable materials in the development of green products [4] because it is the most abundant natural polymer on the Earth. It is also renewable [5], nontoxic [6] and biodegradable [7]. However, its network of intermolecular hydrogen bonds and high stability of the crystalline phase complicate its processing [8]. On the other hand, the outstanding mechanical properties of cellulose crystallites have promoted a growing interest in cellulose nanomaterials with high degrees of crystallinity, such as cellulose nanocrystals [9,10] and nanofibers (CNFs) [11,12]. These materials, also known as nanocellulose or nanocrystalline cellulose, are intensively studied for applications that include composite reinforcement [13,14], biomedicine applications [15,16] and water purification [17,18].

The interest in nanocellulose stems primarily from the fact that its intrinsic architecture involves a combination of high stiffness and strength [19,20]. The ultimate strength of nanocrystals is 7500–7700 MPa and the modulus of elasticity is 110–220 GPa [21]; for CNF these values are 357.5 MPa and 22.9 GPa, respectively [22]. This is the motivation for the current intense studying of the various mechanical and chemical methods for the production of nanocellulose [23]. The processes used for these purposes are mechanical homogenization [24], processing using microfluidizers [25], acid hydrolysis [26], enzymatic hydrolysis [27] and treatment with ionic liquids (ILs) [28]. ILs have the ability to effectively disrupt the intermolecular bonds and dissolve cellulose [29] and they are also used for the isolation of cellulose nanomaterials [30,31].

At the same time, ILs are expensive and not safe for the environment. To avoid these problems, the use of “green” analogs of ILs—deep eutectic solvents (DES)—for cellulose processing has been actively developed in recent years [32]. DES are usually considered to be a class of ILs that demonstrate non-volatility, thermal stability and low toxicity [33]. They are also cheaper than ILs. DES are prepared by mixing two or more components, between which complexation and charge delocalization occur via the formation of hydrogen bonds [34]. The distinctive feature of such solvents is their ability to form an eutectic mixture with a melting point significantly lower than that of each of the individual components [35]. One of the components acts as a hydrogen bond acceptor (HBA); methyltriphenylphosphonium bromide [36] and zinc chloride [37] are examples of such. Choline chloride (ChCl) is the most often used HBA due to its low price and natural origin [38]. Another component of DES—a hydrogen bond donor (HBD)—can be, for example, an organic acid (acetic [39], citric [40], oxalic [41], etc.), polybasic alcohol (ethylene glycol [42], glycerol [43]) or urea [44]. Thus, the number of different combinations of substances that constitute DES is quite large. As a result, the properties of DES can be varied in a controlled manner by changing the type and molar ratio of the components to fit the requirements of the exact practical application.

The application of DES for cellulose processing has attracted much attention. These solvents are intensively studied for the separation of plant cellulose from biomass containing lignin and hemicellulose [45]. DES’s ability to form networks of hydrogen bonds leads to the swelling [46] or even dissolution of cellulose [47]. Recent reviews on cellulose processing with DES and ILs [29,48] report that, in general, ILs are more active in cellulose dissolution than DES. This has been attributed to the less ordered structure of DES in comparison with ILs, and the lack of sufficient entropy gain when a cellulose macromolecule is transferred from the crystalline to the dissolved state [49]. This can be used to facilitate the preparation of nanomaterials [50,51] with the preservation of the initial highly crystalline order of cellulose. For example, in the work of Suopajärvi et al. [52], various waste paper boards were hydrolyzed with ChCl–urea to generate CNFs with width of 2–80 nm. The enhancement of the nanofibrilation of birch cellulose pretreated with glycerol–betaine hydrochloride DES was demonstrated [53]. However, DES have rarely been studied for the extraction of CNFs, except for the cases in which they are applied for the pretreatment of pulp. At the same time, the applicability of DES for the preparation of cellulose nanocrystals has been demonstrated in recent works [54,55]. Additionally, DES are being studied as a media for nanocellulose chemical modification due to their ability to form stable nanocellulose dispersion [56].

Among nanocellulose-based materials, nanopaper has attracted increased attention. This material is defined as a sheet prepared from CNF [57] and it has been proposed to be a material in which the high mechanical properties of nanocellulose can potentially be transferred to the macroscopic level [58]. Recent progress in the field of nanopaper relates to increases in the cost-effectiveness of its production and to its possible applications in composites and flexible electronics. The following three most common directions can be distinguished. Firstly, improving its mechanical properties via methods such as the preparation of aligned CNF paper with extremely high strength (1 GPa of a tensile strength [59]). Second, the possibility of attaining desirable optical properties (transparency, haze and UV-blocking) via the preparation of, for example, hemicellulose- or lignin-containing cellulose nanopaper [60]. Finally, the application of modern approaches for greener cellulose processing with the application of ILs [61] and DES [62] in the routes for the preparation of cellulose nanopaper.

Bacterial cellulose (BC) is a non-wood source of cellulose produced by bacteria, for example, *Acetobacter xylinum* [63]. The advantages of BC include its unique supramolecular structure, which is initially characterized by the presence of a chemically pure network of cellulose nanofibers (BC-NFs) connected via a network of hydrogen-bonds [64]. Bacterial cellulose nanomaterials are widely used in photocatalysis [65], medicine [66], pharmacy [67], the food industry [68], commercial and industrial goods [69] and electronics [70].

In this study, we investigate, for the first time, the treatment of BC with two of the most common and cheapest DES, which are based on choline chloride and ethylene glycol (EG) or glycerol (Gly), and their effect on the structure, morphology, mechanical and thermal properties of prepared BC-NFs. The interest in polyatomic alcohols for the pretreatment of cellulose is due to their ability to form strong hydrogen bonds with this polymer, which makes such alcohols effective plasticizers for the disintegration of intramolecular hydrogen bonds in cellulose [71]. Additionally, its high chemical purity makes BC an interesting model substance for studies into the influence of DES treatment on the cellulose structure, morphology and properties. The cellulose nanopaper based on BC-NFs was also prepared and its thermal and mechanical properties were characterized.

## 2. Materials and Methods

### 2.1. Materials

Choline chloride (CAS 67-48-1, purity >99%, Glentham Life Sciences Ltd., Corsham, UK) was dried before use in vacuum at 60 °C for 24 h. Ethylene glycol (CAS 107-21-1, purity >98%, VEKTON, Saint Petersburg, Russia) and glycerol (CAS 56-81-5, purity >98%, VEKTON, Saint Petersburg, Russia) were used as received. The lyophilized culture of *A. xylinum* was purchased from the All-Russian collection of industrial microorganisms (National Bioresource Center, GosNIIgenetics, Moscow, Russia) and cultured as described in Section 2.2. Peptone and D-mannitol (CAS 69-65-8) were obtained from LenReaktiv (Saint Petersburg, Russia), yeast extract from the Research Center for Pharmacotherapy (Saint Petersburg, Russia) and NaOH (CAS 1310-73-2) from NevaReaktiv (Saint Petersburg, Russia). Sodium tartrate dehydrate (CAS 868-18-8, purity >99.5%) and anhydrous iron(III) chloride (CAS 7705-08-0, purity >99.5%) were purchased from NevaReaktiv (Saint Petersburg, Russia) and Sigma-Aldrich (St. Louis, MO, USA), respectively.

### 2.2. Methods

#### 2.2.1. Bacterial Synthesis of Cellulose

Lyophilized *A. xylinum* was activated by introducing a seed culture medium. The colony of *A. xylinum* was transferred into 400 mL of seed culture medium, and then cultured under static conditions for 3 months at a temperature of 27 °C. After that, 200 mL of cell suspension was introduced into 1 L of culture medium, which contained 0.3 wt% of peptone, 0.5 wt% of yeast extract and 2.5 wt% of D-mannitol. The medium was incubated statically at 27 °C for 15–20 days in an incubator (BINDER, Tuttlingen, Germany) until a 1 cm thick bacterial cellulose film was formed. The bacterial cellulose membrane was purified via boiling in a 0.5% NaOH solution for about 30 days and then thoroughly washed with distilled water until it reached pH 7. The obtained cellulose gel-film was ground with a laboratory blender, SteglerLB-2 (Ningbo Yilin Electric Appliance Co, Ningbo, China), and then lyophilized with a Scientz-10ND lyophilic dryer (Scientz, Ningbo, China) in Petri dishes. The obtained porous bacterial cellulose aerogels were crushed to pieces with sizes in the range of 1–3 mm that was used for further treatment with DES.

#### 2.2.2. Preparation of Deep Eutectic Solvents

ChCl was mixed with alcohols (EG or Gly) at a molar ratio of 1:2 in a flask equipped with a magnetic stirrer and a tube with CaCl_2_. For the preparation of 10 g ChCl/EG DES, 5.3 g of ChCl and 4.2 mL of EG were mixed. In the case of ChCl/Gly DES, the mass of ChCl was 4.3 g, and 4.5 mL of glycerol was taken. The mixtures were heated up at 60 °C using an oil bath until they became homogeneous.

#### 2.2.3. Treatment of BC with DES and Preparation of BC-NF Suspension

BC (0.1 g) was added to the DES and mixed until the formation of a homogeneous viscous suspension occurred. The prepared systems were stirred for 90 min at 65 °C. In the rest of the text, samples prepared with ChCl/EG and ChCl/Gly will be denoted as E and G, respectively. Both systems were prepared either with ultrasonication (samples E+ and G+) or without (samples E− and G−). An ultrasonic bath (Sapphire 2.8L TTC (RMD), Moscow, Russia) with 100 W power was used. In the next step, all the samples were diluted with 40 mL of ethanol and the mixture was centrifuged for 30 min at a speed of 8000 rpm (Digisystem Laboratory Instruments Inc., New Taipei City, Taiwan). The liquid upper phase was removed via decantation and the residual was redispersed with 40 mL of water and centrifuged again. The centrifugation and redispersion procedures were repeated at least 10 times to remove the DES residues. As a result, viscous bacterial cellulose nanofiber hydrogel was obtained. The concentrations of the dispersions were measured through the drying of about 5 g of gel in an oven at 110 °C with the subsequent weighing of the remaining cellulose nanofibers using an AP225WD (Shimadzu, Kyoto, Japan) microbalance with an accuracy of 0.01 mg.

#### 2.2.4. Preparation of Cellulose Films

**Figure 1:**
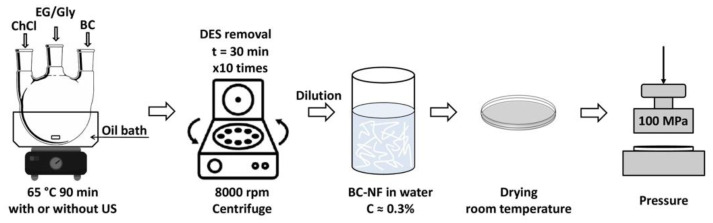
Sample preparation scheme of cellulose films processed in the ChCl/EG and ChCl/Gly mixtures.

The obtained dispersions were diluted with water in order to attain a 0.3 wt% concentration of BC-NFs. They were put in a Petri dishes and dried at room temperature until a constant mass was reached. The self-standing films were separated from the Petri dish and pressed for 2 h under a load of 100 MPa using the AG-100kNX Plus mechanical testing machine (Shimadzu, Kyoto, Japan), equipped with a compression module. The overall scheme of the sample preparation is given in Figure 1.

The compressed BC-NF films were used to determine the paper density and degree of polymerization (DP). To determine the density, the film was weighed using an AP225WD (Shimadzu, Kyoto, Japan) microbalance and its thickness was measured. The density of the compressed film was determined by the ratio of the mass of the sample to its volume, calculated using the diameter of the film. The DP of the samples was determined from the characteristic viscosity [η] of the BC or BC-NF solutions in iron(III) sodium tartrate using the equation $\left[\eta \right]=2.74\times 10^{-2}{\;\mathrm{DP}}^{0.775}$ [72].

#### 2.2.5. Infrared and Raman Spectroscopy

An attenuated total reflection (ATR)-FTIR spectroscopy study was performed using the IRAffinity-1S spectrometer (Shimadzu, Kyoto, Japan) with 100 scans at resolution 2 cm^−1^ from 3900 to 600 cm^−1^. The GS10800-B (Specac, Orpington, UK) ATR compartment that allows the user to provide a constant clamping force was used. ATR correction was applied to the spectrum using the LabSolutions v.1.50 software (Shimadzu, Kyoto, Japan).

Raman spectra were obtained with the confocal Raman microscope alpha300R (WITec GmbH, Ulm, Germany). The microscope objective lens of 50× and the excitation line 633 nm of a He-Ne laser give a spatial resolution of ~1.0 μm. Raman maps of some characteristic bands were obtained for an area of 30 × 30 μm^2^, considering 30 points per line and 30 lines, and 5.0 s of exposition time at each point. Data processing was carried out using the WITec Project FIVE software (version 5.1, WITec GmbH, Ulm, Germany).

#### 2.2.6. Wide-Angle X-ray Diffraction Study

The crystalline structures of the films were studied via wide-angle X-ray diffraction (WAXD) with a Rigaku SmartLab 3 diffractometer (Rigaku Corporation, Tokyo, Japan) equipped with a CuK_α_ radiation source (λ = 1.54 Å) within the 2θ range of 5°–40° with the scan step of 0.05°. The crystallinity index (CrI) was calculated using the empirical method suggested by Segal et al. [73].

#### 2.2.7. Microscopic Investigation

Atomic force microscopy (AFM) studies were performed using the SPM-9700HT scanning probe microscope (Shimadzu, Kyoto, Japan) operating in the tapping mode, with 256 points per line and 256 lines per image. NSG30-SS silicon cantilevers, provided by TipsNano, were used, and the nominal resonant frequency was 340 kHz, the hardness coefficient was 40 N/m and the probe radius was 2 nm. Experimental data were processed using the SPM software v.4.76.1 (Shimadzu, Kyoto, Japan) software. Scanning electron microscopy (SEM) images of the films’ surfaces were obtained with a Tescan Vega III microscope (Tescan, Brno—Kohoutovice, Czech Republic) at 10 kV voltage. The samples were covered with a carbon layer by a rotary pumped coater Quorum Q150R E (Quorum, West Sussex, UK).

#### 2.2.8. Thermogravimetric Analysis (TGA)

The thermogravimetric analysis was performed with the use of a DTG-60 setup (Shimadzu, Kyoto, Japan). The samples were heated at the rate of 5 °C/min in argon flow (80 mL/min). Extrapolated onset temperature, T_onset_ extrapolated, for the mass-loss step on the TGA curves was determined as an intersection of the tangents to the TGA curve at the points of maximum and minimum gradients according to the ISO 11358-1 standard. The TGA curves obtained were also used to determine the τ_5_ values (the temperature at which the polymer loses 5% of its initial weight due to the thermal destruction processes).

#### 2.2.9. Mechanical Measurements

The mechanical characteristics of the film samples were investigated using an AGS-X 5 kN setup (Shimadzu, Kyoto, Japan) operating in the uniaxial extension mode. The ambient conditions during the mechanical measurements were 22–23 °C and 40–45% of relative humidity. Strip-like samples (2 × 15 mm^2^) were stretched at room temperature at a rate of 10 mm/min. Young’s modulus (E), the break stress (σ) and the ultimate deformation (ε) were determined.

## 3. Results

### 3.1. Preparation of Dispersions and Films

**Figure 2:**
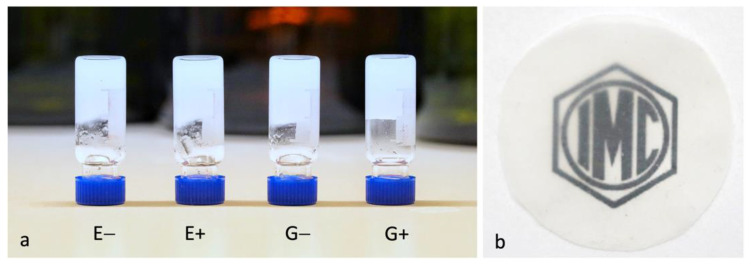
Photos of BC-NF dispersions prepared under different conditions (**a**) and example of dried BC-NF paper (**b**).

Figure 2a shows photos of the BC-NF suspensions in water. The morphologies of the samples were determined using AFM, and are presented in Section 3.4. The dispersions were diluted, casted and dried for the formation of films (Figure 2b). All further tests were performed on BC-NF films.

Table 1 shows the characteristic viscosity [η], the DP values of the initial BC and BC-NFs and the density of the BC-NF paper obtained under various processing conditions. The results demonstrate decreasing [η] and, consequently, a DP in all cases indicating the depolymerization of the cellulose in the selected types of DES. It is seen from Table 1 that ultrasonic treatment leads to a more pronounced decrease in the DP for both DES. In addition, the processing of BC with ChCl/EG leads to lower values of DP compared to the values found for the samples treated with ChCl/Gly. The BC-NF paper density data are in the range of 1.18–1.24 g/cm^3^, which is comparable to the examples of nanopaper from the literature, and have typical densities of 0.9–1.5 g/cm^3^ [74,75,76]. In addition, it is worth noting that the processing method in our case does not significantly affect the value of the density. However, the slightly higher density for the samples prepared with the application of ultrasound was found in both cases.

### 3.2. Infrared and Raman Spectroscopy

**Figure 3:**
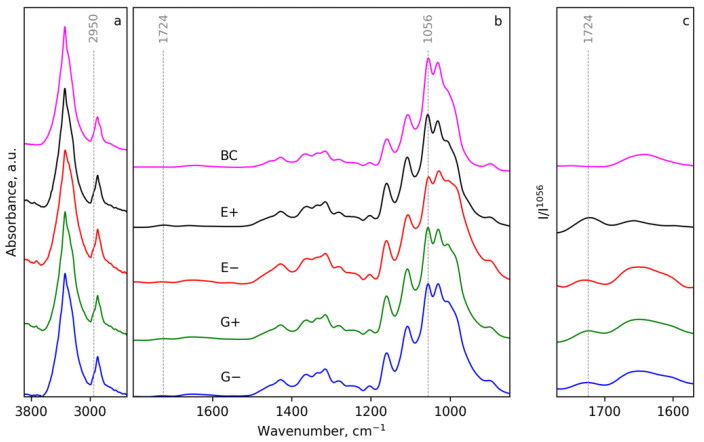
FTIR spectra of initial BC and BC-NFs prepared under different conditions in regions 3900–2500 cm^−1^ (**a**), 1800–850 cm^−1^ (**b**) and 1780–1570 cm^−1^, normalized with the intensity of absorption at 1056 cm^−1^ (**c**).

FTIR spectra were measured for the initial BC and BC after treatment with DES both with and without applying ultrasound. Spectra in the region of 1800–850 cm^−1^ (Figure 3b) demonstrate that, in general, the typical absorption bands of BC are preserved at the same positions. Thus, the chemical structure of the bulk of cellulose is not significantly affected in any of the cases. However, a more detailed presentation of the region 1770–1560 cm^−1^ (Figure 3c) shows the appearance of new band centered near 1724 cm^−1^. Considering the literature data on absorption in the carbonyl group region of cellulose [77], the band at 1724 cm^−1^ can be attributed to the aldehyde groups. This idea is supported by the results regarding the DP of the cellulose that demonstrate a decreasing DP during DES treatment. Additionally, the stronger depolymerization under the application of ultrasound (samples E+ and G+) correlates with the higher intensity of band 1724 cm^−1^ in the spectra, normalized on absorption at 1056 cm^−1^ (Figure 3c). In the region of 3900–2500 cm^−1^, slight changes can be observed (Figure 3a) in the shape of both the absorption bands centered near 2890 and 3340 cm^−1^. They correspond to the C-H and O-H vibrations, respectively. The appearance of a pronounced shoulder near 2950 cm^−1^ can be attributed to the changing of the C-H bound vibrations that can be related to changes in the neighbouring chemical structures due to the disruption of the glycosidic links.

**Figure 4:**
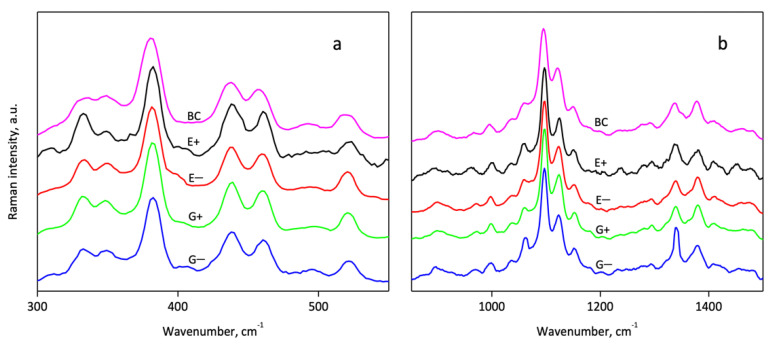
Raman spectra of initial BC and BC-NFs prepared under different conditions in the regions 300–550 cm^−1^ (**a**) and 850–1500 cm^−1^ (**b**).

Additional data on the structure of the BC-NFs prepared with different conditions were obtained using Raman spectroscopy. The spectra given in Figure 4a,b also confirm the preservation of the cellulose chemical structure in general. An analysis of the ratios of some bands gives additional information about the structural features of the cellulose samples. Thus, the ratio of intensities between bands at 1121 and 1096 cm^−1^ (I^1121^/I^1096^) was attributed to the level of organization of the fibers: The intensity of the 1121 cm^−1^ band decreases with the fission of the interchain linkages [78]. In the case of the studied BC-NFs, the maxima of these bands were found at 1123 and 1097 cm^−1^. The ratio I^1123^/I^1097^ was 0.65, 0.59, 0.55, 0.58 and 0.59 for BC, E− E+, G− and G+ samples, respectively. This indicates the increased disordering of the surfaces of the samples prepared with ethylene glycol-based DES in combination with ultrasound. Additionally, it is interesting to evaluate the spatial distribution of the disorder of the films’ surfaces with the mapping of the I^1123^/I^1097^ ratio using Raman microscopy. Maps demonstrating the normalized values of the I^1123^/I^1097^ ratio at different points of the surface are shown in Figure S1 It is seen that the deviation of the I^1123^/I^1097^ value is observed for all samples: areas with higher ordering (light) are surrounded by regions with lower ordering (dark areas). It can be noticed that the range of variation of I^1123^/I^1097^ on the maps is lower for the samples prepared with the application of ultrasound (E+ and G+) in comparison with the samples prepared without it (E− and G−). Thus, for ultrasound-treated samples, a more uniformly disordered BC-NF surface structure can be proposed, while for other samples the more pronounced variation of the degree of the polymer chain ordering along the surface can be assumed.

### 3.3. WAXD Data

**Figure 5:**
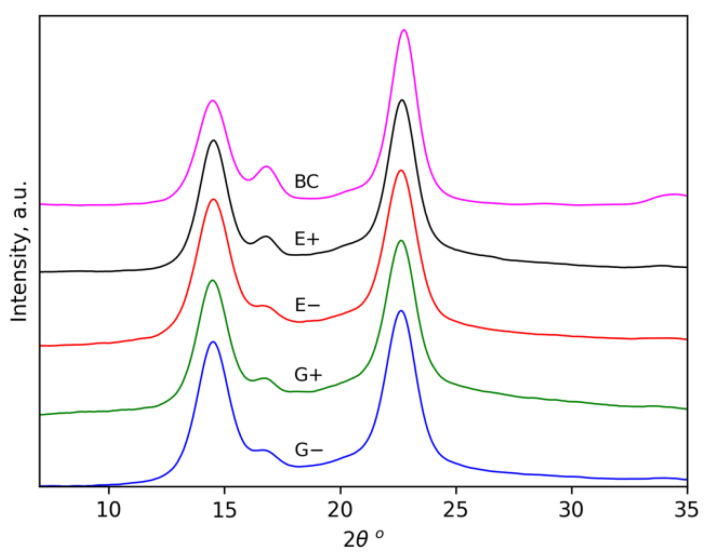
X-ray diffraction patterns of initial BC and BC-NF samples prepared under different conditions.

X-ray diffraction was carried out to compare the crystalline structure of the cellulose films. X-ray patterns are presented in Figure 5. All the samples exhibited similar patterns with main characteristic peaks at 2θ angles of approximately 14.5°, 16.7° and 22.7°, which represent the typical peaks of cellulose Iα structure reflections and can be attributed to the (100), (010) and (110) planes, respectively [79]. In order to evaluate the effect of ultrasonication on the crystal structure, the crystallinity index (CrI) values of the samples were calculated according to the Segal method [73]. The CrI values for E−, E+, G− and G+ were 89.5 ± 0.5%, 89.0 ± 1.5%, 87.9 ± 0.7% and 86.1 ± 1.5%, respectively. All of them are slightly lower in comparison to the initial BC (95 ± 1%), and this agrees with other results demonstrating the partial disordering of cellulose during treatment in DES. The degree of crystallinity determined does not demonstrate significant differences between the samples treated in different conditions. The cellulose polymorph Iα is preserved during the treatment (a change in the crystalline structure was not observed). This is expected for cellulose treatment with DES because most of them, in contrast to ILs, are unable to dissolve cellulose [48]. This allows us to propose that the bulk of isolated BC-NFs under the selected conditions of treatment remains untouched. It can be suggested that the main difference between the BC-NFs prepared in different conditions is connected to variations in the structures of their surfaces.

### 3.4. Microscopy Investigation

**Figure 6:**
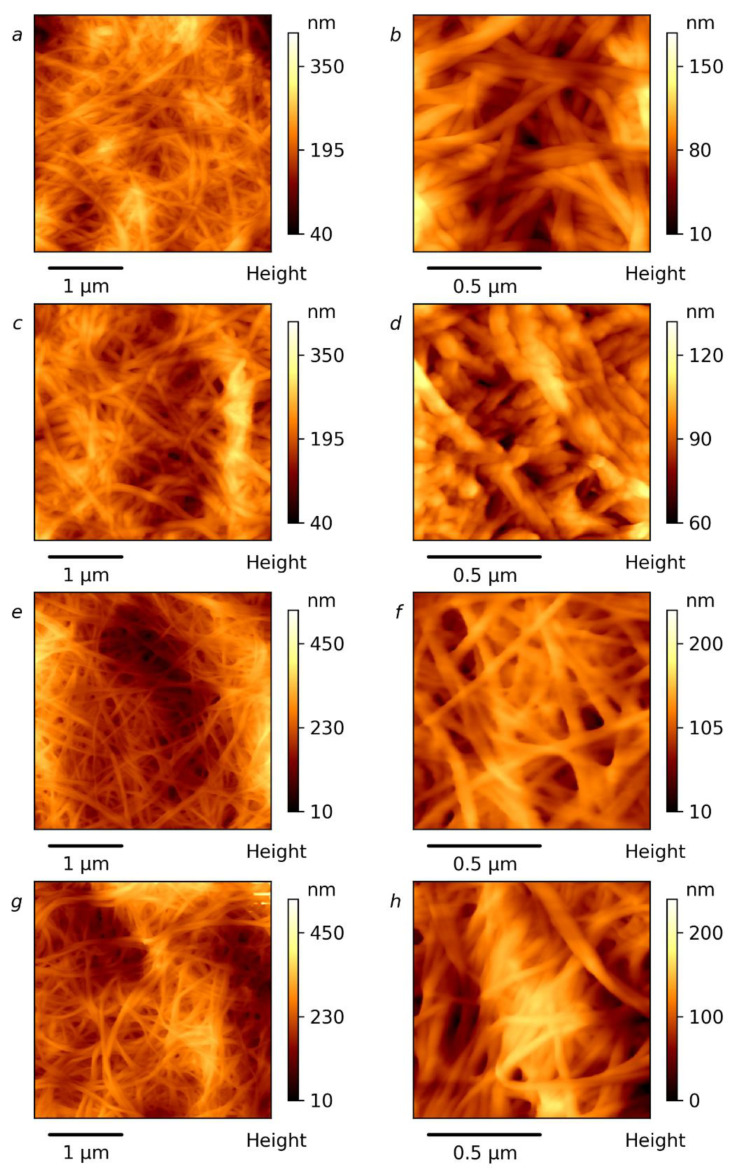
AFM images of surface topography of BC-NFs: E− (**a**,**b**), E+ (**c**,**d**), G− (**e**,**f**) and G+ (**g**,**h**).

The morphologies of the prepared BC-NFs were studied using AFM and the results are given in Figure 6 and Figure S1. It can be seen that the BC-NFs are well-individualized and can easily be observed. The widths of the BC-NFs after different treatments were determined from about 100 measurements using AFM maps (see Figure S2). The mean diameter of the nanofibers in the initial BC is 27 nm and the overall distribution of the diameters can be seen in the histograms in Figure S3. It is obvious that distributions in all cases move toward higher dimensions during treatment with DES, demonstrating a swelling of cellulose. The AFM image for the BC-NFs without ultrasonication treatment (E−) shows a smooth, web-like network structure and occurs as very long entangled filament-like nanosized fibers (Figure 6a,b). The surfaces of the BC nanofibers with ultrasonication treatment (E+) become, however, irregular in comparison to the E− sample. This morphological change during ultrasonic treatment may be connected to the decreasing DP of the cellulose on the surface layers and its possible swelling, which is more pronounced in the case of the ultrasound. The corresponding width distributions are given in Figure S3, and they were used for the calculation of the average diameters of the BC-NFs. The average diameters of 44, 55, 41 and 44 nm were found for the E−, E+, G− and G+ samples, respectively. In the case of ethylene glycol-based DES, the distribution demonstrates the most pronounced shifting toward higher diameters, and thus the most intensive swelling of the BC-NFs can be proposed for these conditions. Morphological analysis for the samples prepared using glycerol-based DES revealed that the nanofibrils of the G− and G+ samples had approximately similar mean diameters, demonstrating the lesser influence of ultrasound on this parameter. At the same time, in the case of the G+ sample, the flattening of the maximum of the distribution can be seen. This can be attributed to the competition between the process of splitting the BC-NFs and the decrease in their diameter accompanied by the swelling of the BC-NFs that results in an increase in the fibers’ diameters. The swelling of the BC-NFs under the application of ultrasound agrees with the decreasing DP and the increase in the nanofibers’ surface roughness that can relate to the destruction of the cellulose packaging on the fibril surfaces. The enhanced swelling of the cellulose fibers under the application of ultrasound is in agreement with previously reported data on the treatment of cellulose with an aqueous N-methyl-morpholine-N-oxide solution [80].

**Figure 7:**
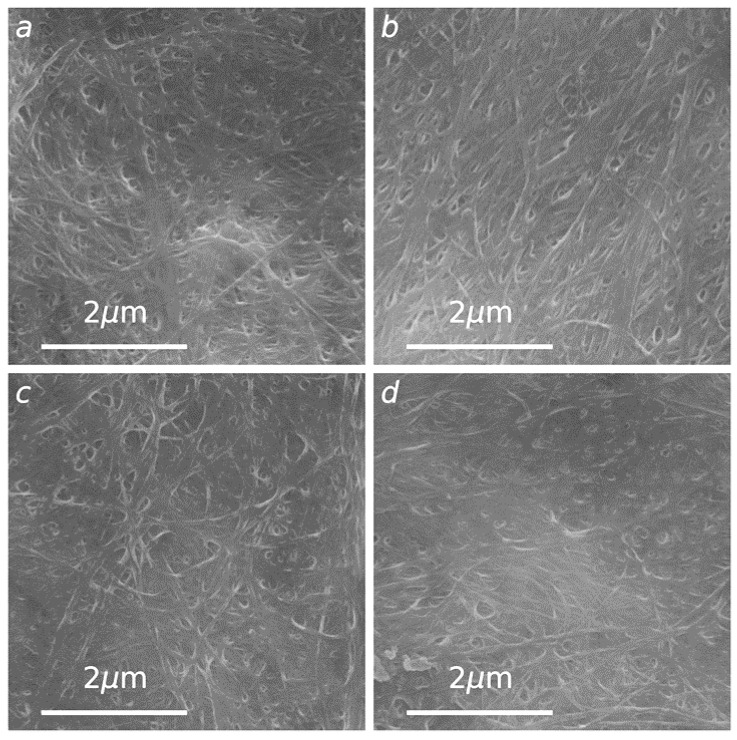
SEM images of cellulose films: E− (**a**), E+ (**b**), G− (**c**) and G+ (**d**).

To obtain more insights into the prepared BC-NF film structures, SEM images of the films’ surfaces were acquired (Figure 7). As shown in Figure 7, the surfaces of all of the films had a compact cellulose network-like structure consisting of ultrafine nanofibers resembling randomly entangled ribbons. In addition, the SEM data demonstrate that all films are dense and there is no significant difference in fiber packing density. This data correlate with the BC-NF paper density data discussed earlier (see Table 1).

### 3.5. Thermogravimetric Analysis

**Figure 8:**
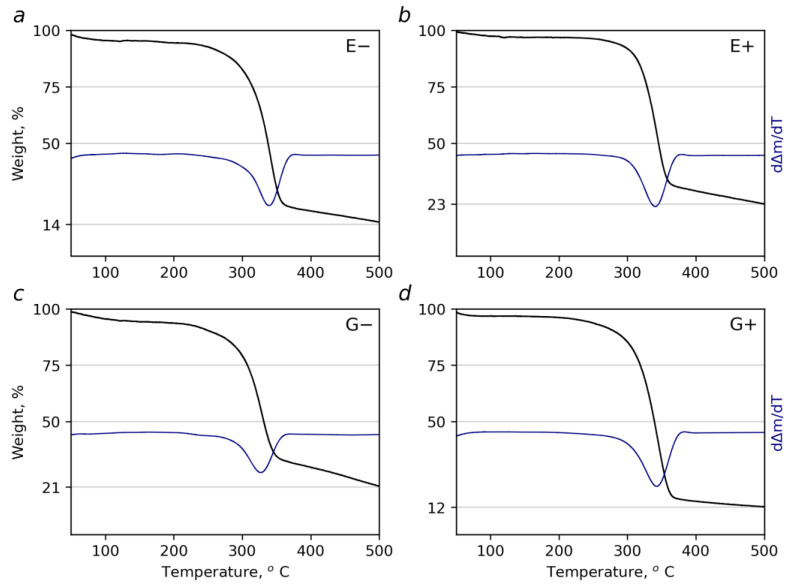
Dependences of weight loss (in %) and dΔm/dT on temperature of BC-NFs prepared under different conditions: E− (**a**), E+ (**b**), G− (**c**), G+ (**d**).

When heated up to ~200 °C, the samples lost 3.0–6.3% of their initial mass (Figure 8, Table 2). This step corresponds to the removal of the adsorbed water. It is seen that papers prepared from ultrasound-treated BC-NF contain lower amounts of water. This can be related to the slightly higher density of these samples (see Table 1), which reflects lower free space inside the papers and, therefore, lower moisture absorbance. At higher temperatures, two stages of cellulose decomposition were observed. The first mass loss (in the temperature interval of 200–370 °C) is the most pronounced and is brought about by two concurring processes involving the dehydration and depolymerization of cellulose [81]. The corresponding T_onset_ extrapolated values as well as the τ_5_ values are summarized in Table 2. The next stage continuing up to 800 °C is attributed to the slow degradation of the organic residue. The thermal stability of almost all of the samples is comparable with that of the initial BC (Table 2, Figure S4). Table 2 contains literature data on the thermal properties of CNF prepared with the use of other methods. It is seen that the samples obtained in this work possess better thermal stability. The slightly higher thermal stability (higher T_onset_ values) can be noticed for the BC-NF prepared with the application of ultrasound. This can also be attributed to the slightly higher density of these samples that results in the lower permeability, restricting the elimination of decomposition products.

### 3.6. Mechanical Measurements

**Figure 9:**
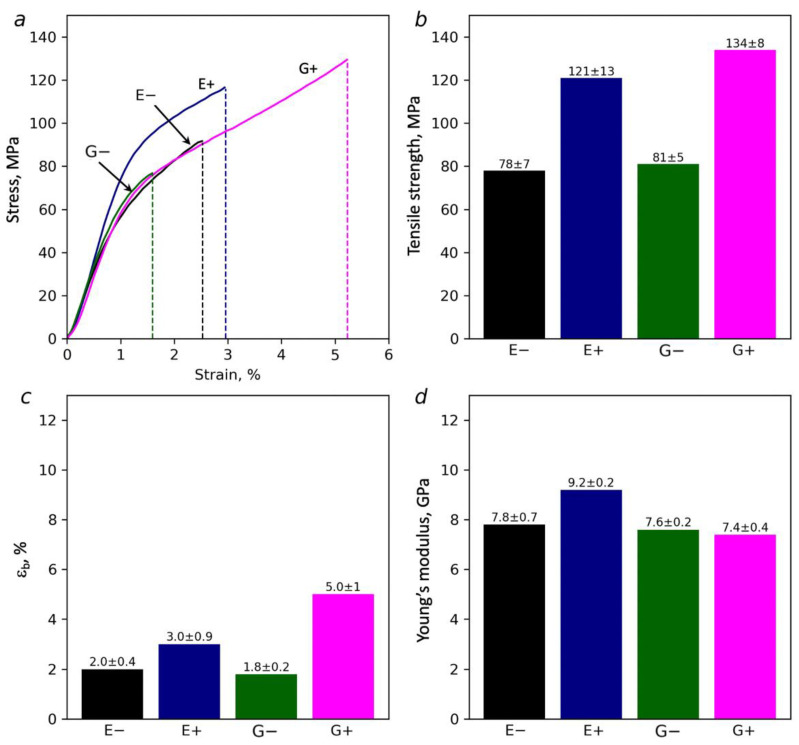
Stress-strain curves for the BC-NF films prepared under different conditions (**a**) and corresponding values of tensile strength (**b**), elongation at break (**c**) and Young’s modulus (**d**).

The results of the mechanical tests on the paper samples demonstrate differences in their behaviors under mechanical stress. Figure 9 clearly shows that the treatment of the dispersions by the ultrasound causes an improvement in the mechanical strengths. Strength increases from 78 up to 121 MPa and from 81 up to 134 MPa for the BC-NFs prepared with EG and Gly-based DES, respectively. Moreover, the strength of the ultrasound-treated samples in the presence of DES is higher than the 96 MPa that was reported earlier for the initial BC [85]. The elongation at break for the samples prepared with ultrasound is also higher. This correlates with the more uniform structural disordering of the surface of the BC-NF paper prepared with ultrasound that was proposed based on the Raman study. Considering the structural data discussed above, it was suggested that ultrasound contributes to the destruction (disordering) of the surface layer due to cellulose depolymerization. In the case of the E+ sample, this is also supported by Raman spectroscopy. At the same time, the structure of the bulk of the fibers preserves high crystallinity, as was demonstrated with the WAXD data. It can be proposed that surface disordering can lead to the appearance of an increased number of accessible -OH groups on the surface of the BC-NFs. This can result in an increased adhesion between the BC-NFs and, consequently, an increase in the strength of the interfibrillar contacts. This is also supported by the slightly higher densities of the BC-NF papers prepared with the application of ultrasound. Finally, the better interaction between the BC-NFs contributed to the improvement of the mechanical characteristics.

Additionally, it can be suggested that pretreatment with DES in combination with ultrasound better dissolves the amorphous parts of the cellulose due to increased penetration of DES into the cellulose and the improved mass transfer [86,87]. As a result, it is likely that the stronger cellulose residues remain, and that it is due to these parts that the stronger and stiffer samples are formed. The maximal effect of the combination of ultrasound and DES treatment observed for the E+ sample can be attributed to the following: due to the smaller size of the ethylene glycol molecule, it penetrates faster inside the cellulose and better facilitates the destruction of amorphous parts compared to glycerol; in the case of the paper obtained using ethylene glycol and ultrasound, the maximum Young’s modulus of 9.2 GPa was observed, whereas for glycerol it was 7.4 GPa. Finally, the elastic modulus for all films prepared using DES treatment in this work was higher than the value of 6.41 GPa that has been reported for pure BC [85].

Along with the TGA data, the improvement of mechanical characteristics after the ultrasound treatment is associated with a lower water content (Table 2). Water is a plasticizer that can reduce the strength of the connections between the individual BC-NFs, and its presence can lead to a reduction in strength and to a lower Young’s modulus. This is confirmed by the mechanical characteristics of the films (Figure 9).

To conclude, the mechanical characteristics, such as Young’s modulus and tensile strength, of the BC-NFs prepared via treatment with DES combined with ultrasound were higher than the characteristics reported for pure BC [85]. It was higher than the 2.7 GPa and 98 MPa reported for the acidic DES-pretreated ramie nanofibers [62] and comparable with 12.2 GPa and 134 MPa reported for the BC nanopaper prepared using high compaction force (1 t) at 120 °C [88].

## 4. Conclusions

In this study, BC-NF films obtained from dispersions prepared through the treating of lyophilized bacterial cellulose with DES systems (ChCl/EG and ChCl/Gly) both with or without the application of ultrasound were investigated. It was found out that the samples treated with ultrasound and the DES system with ethylene glycol had the best mechanical characteristics. The Young’s modulus of this sample was 9.2 GPa and the strength was 121 MPa. These data correlate with the proposed theoretical concepts about the effect of DES compositions and ultrasound treatment on the morphology and structure of BC-NFs. Indeed, the size of the ethylene glycol molecule is smaller than that of the glycerol molecule and, consequently, the DES, which included ethylene glycol, can diffuse more easily into the cellulose structure and induce the formation of a disordered surface layer. Exposure to ultrasound additionally contributed to the depolymerization of the surface layer and further increased its disorder. The FTIR and AFM data confirm the above findings. The results presented here have some interesting implications for future studies of the application of BC-NFs for the preparation of 1D (fibers), 2D (papers sheets) or 3D (bulk composites) materials. Particularly, they show that more attention to the influence of CNF or BC-NF preparation conditions on their surface structure and, consequently, the strength of interfiber or CNF–polymer matrix interaction is needed. Potentially, this can open a new way for transferring the excellent mechanical properties of highly crystalline cellulose (that is preserved in the case of DES-based treatment) onto the macroscopic level.

## Figures and Tables

**Table 1:** Characteristic viscosity and degree of polymerization (DP) of the initial BC and BC after DES treatment.

Sample	Characteristic Viscosity, [η] (dL/g)	DP	Density of Paper (g/cm^3^)
BC	9.1 ± 0.7	1800 ± 200	–
E−	4.5 ± 0.2	740 ± 50	1.18 ± 0.02
E+	3.9 ± 0.1	610 ± 30	1.20 ± 0.03
G−	7.6 ± 0.3	1410 ± 70	1.22 ± 0.01
G+	5.6 ± 0.5	970 ± 120	1.24 ± 0.03

**Table 2:** Thermal properties of BC-NFs prepared under different conditions.

Sample	T_onset_, °C	τ_5_, °C	Mass Loss at Low Temperature Step, %
E−	309 ± 3	273 ± 16	5.3 ± 0.4
E+	318 ± 3	300 ± 18	3.0 ± 0.2
G−	302 ± 3	270 ± 16	5.1 ± 0.4
G+	312 ± 3	285 ± 17	3.3 ± 0.3
BC	319 ± 3	275 ± 17	4.0 ± 0.3
TOCN TEMPO-mediated oxidation [82]	-	200	-
CNFs-H sulfuric acid hydrolysis [83]	274	-	-
F-CNF formic acid hydrolysis with high-pressure homogenization [84]	291.24	-	-

## Data Availability

Data are available within the article or its Supplementary Materials.

## Supplementary Materials

The following are available online at https://www.mdpi.com/article/10.3390/polym14010078/s1, Figure S1: Raman maps of the intensity ratios of between the bands 1121 cm^−1^ and 1096 cm^−1^ of BC-NF films prepared in different conditions: E− (a), E+ (b), G− (c) and G+ (d), Figure S2: AFM maps with measured BC-NF diameters for samples E− (a), E+ (b) G− (c) and G+ (d), Figure S3: Histograms for distributions of BC-NF diameters for samples E− (a), E+ (b) G− (c) and G+ (d), Figure S4: TGA curve for initial BC.
