# Supplementary material for: Strengthening Cellulose Nanopaper via Deep Eutectic Solvent and Ultrasound-Induced Surface Disordering of Nanofibers
Source: Polymers (Basel). 2021 Dec 26;14(1):78. doi: 10.3390/polym14010078 (PMC8747671; doi:10.3390/polym14010078)
Supplement: Supplementary file 1 [file polymers-14-00078-s001.zip › polymers-1499862-supplementary.pdf]

Polymers

ELECTRONIC SUPPLEMENTARY INFORMATION FOR

STRENGTHENING OF CELLULOSE NANOPAPER VIA DEEP EUTECTIC SOLVENT AND ULTRASOUND-INDUCED SURFACE DISORDERING OF NANOFIBERS

Elizaveta V. Batishcheva[1,2], Darya N. Sokolova[1,2], Veronika S. Fedotova[1], Maria P. Sokolova[1], Alexandra L. Nikolaeva[1], Alexey Y. Vakulyuk[1], Christina Y. Shakhbazova[1,2], Mauro Carlos Costa Ribeiro[3*], Mikko Karttunen[1,4,5,6*], Michael A. Smirnov[1*]

[1] Institute of Macromolecular Compounds, Russian Academy of Sciences, V.O. Bolshoi pr. 31, 199004 St. Petersburg, Russia; batischevaelisaveta@gmail.com (E.V.B.); darya.sokolova.2014@mail.ru (D.N.S.); fedotova.veronicka2016@yandex.ru (V.S.F.); pmarip@mail.ru (M.P.S.); alexandra.l.nikolaeva@gmail.com (A.L.N.); hristka11@yandex.ru (Ch.Ya.Sh.)

[2] Institute of Chemistry, Saint Petersburg State University, Universitetsky pr. 26, Peterhof, 198504 Saint Peters-burg, Russia

[3] Departamento de Quìmica Fundamental, Instituto de Quìmica, Universidade de São Paulo, Avenida Professor Lineu Prestes 748, São Paulo, 05508-000 Brasil

[4] Department of Chemistry, The University of Western Ontario, 1151 Richmond Street, London, ON N6A 5B7, Canada; mkarttu@uwo.ca (M.K.)

[5] Department of Physics and Astronomy, The University of Western Ontario, 1151 Richmond Street, London, ON N6A 5B7, Canada

[6] The Centre of Advanced Materials and Biomaterials Research, The University of Western Ontario, 1151 Richmond Street, London, ON N6A 5B7, Canada

* Correspondence: Smirnov_Michael@mail.ru; mkarttu@uwo.ca; mccribei@iq.usp.br

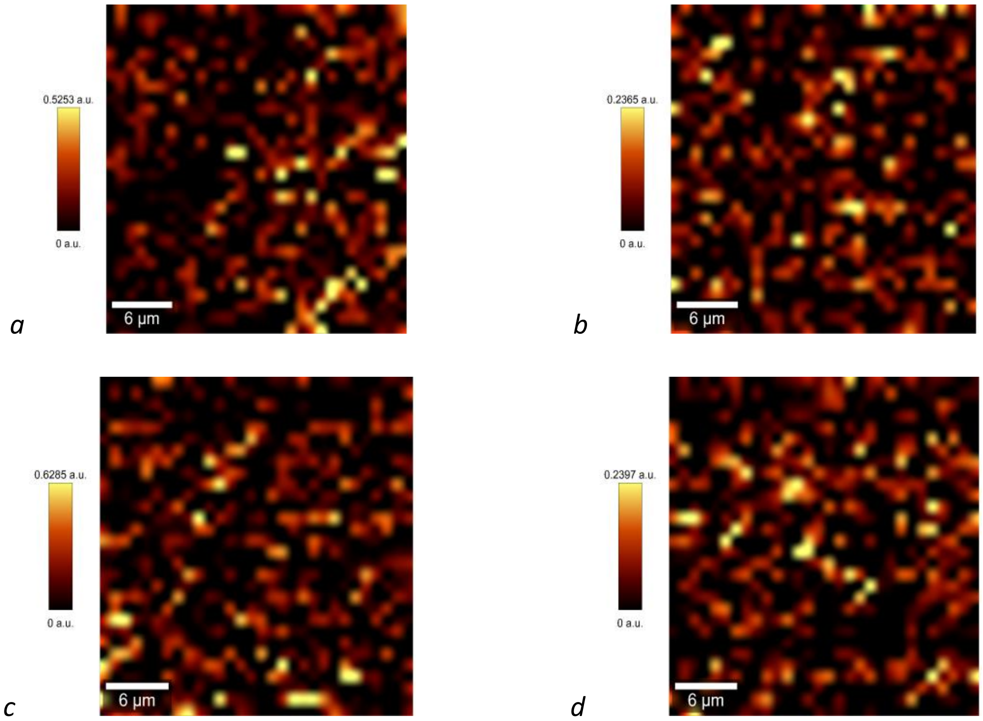

Figure S1. Raman maps of the intensity ratios between the bands 1123 cm^{-1} and 1097 cm^{-1} of BC-NF films prepared under different conditions: E– (a), E+ (b), G– (c) and G+ (d).

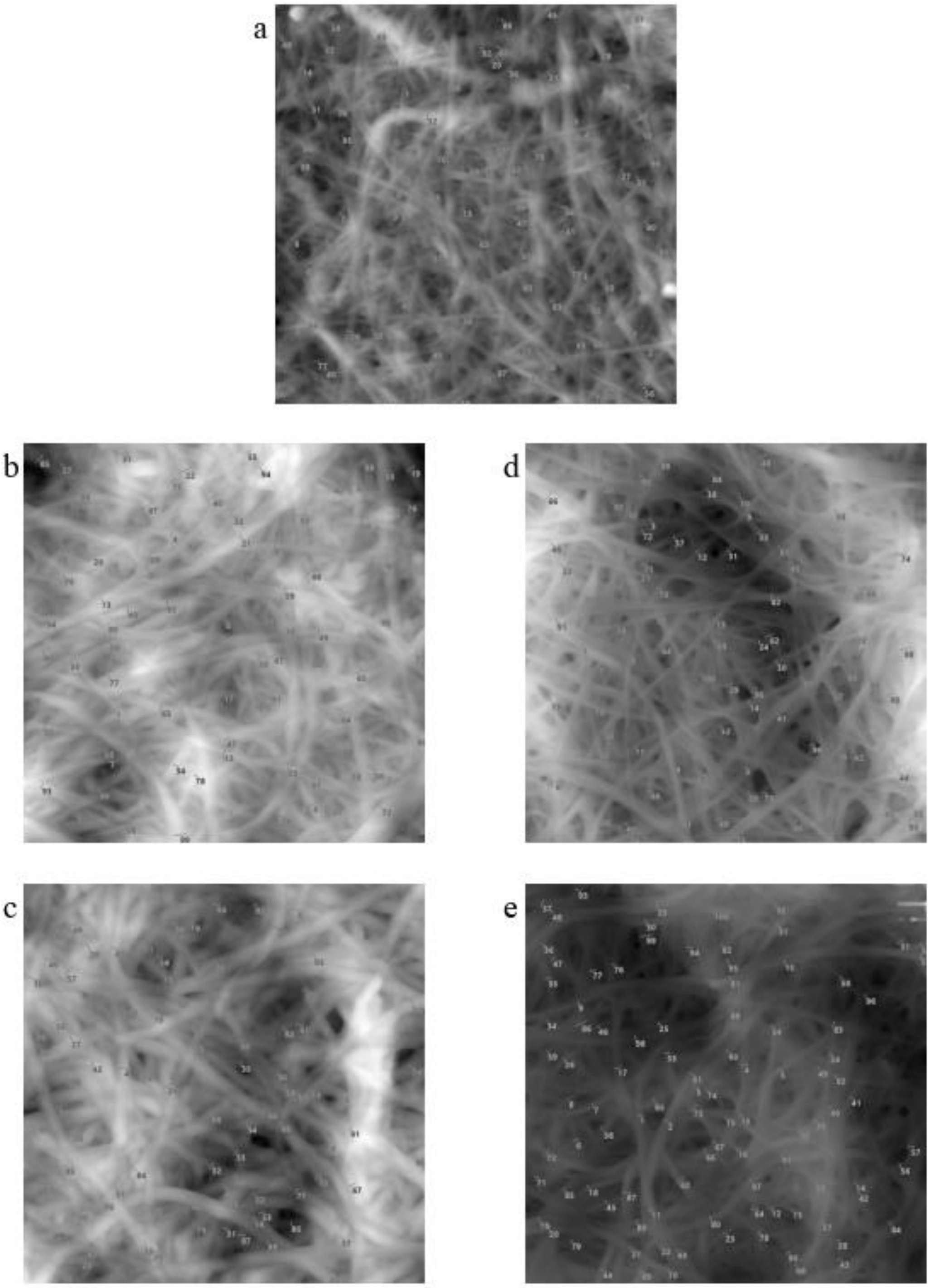

Figure S2. AFM maps of BC-NF with width measurements: initial BC (a), E– (b), E+ (c), G– (d) and G+ (e).

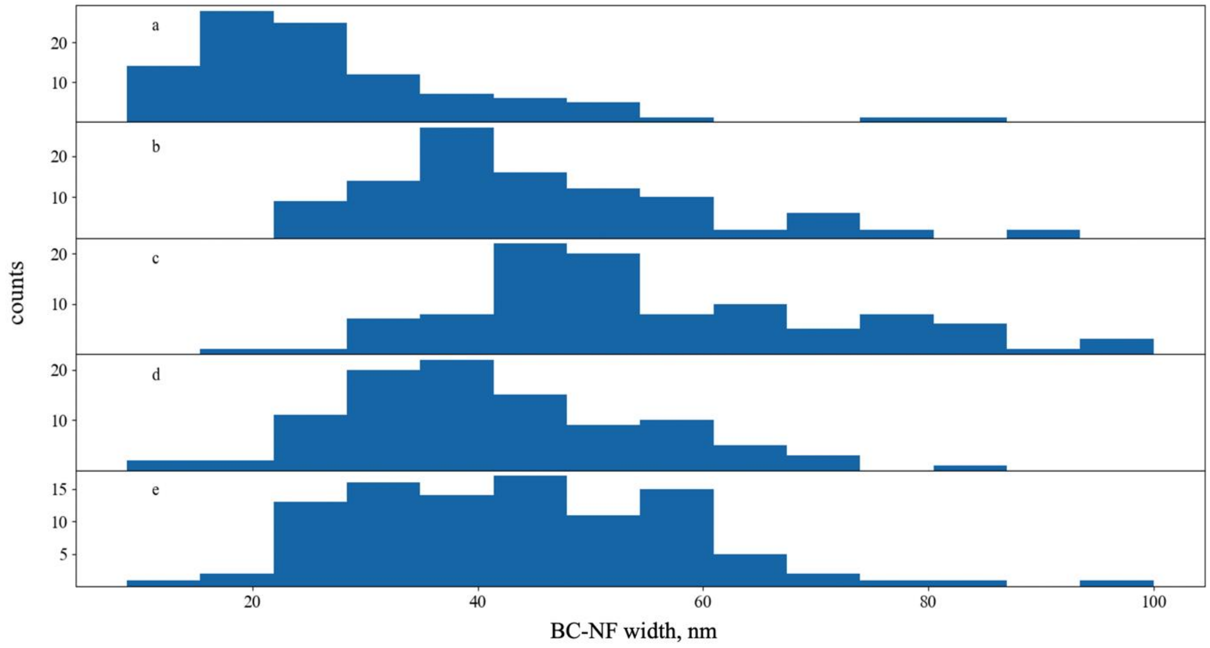

Figure S3. Histograms of BC-NF width distributions: initial BC (a), E– (b), E+ (c), G– (d) and G+ (e).

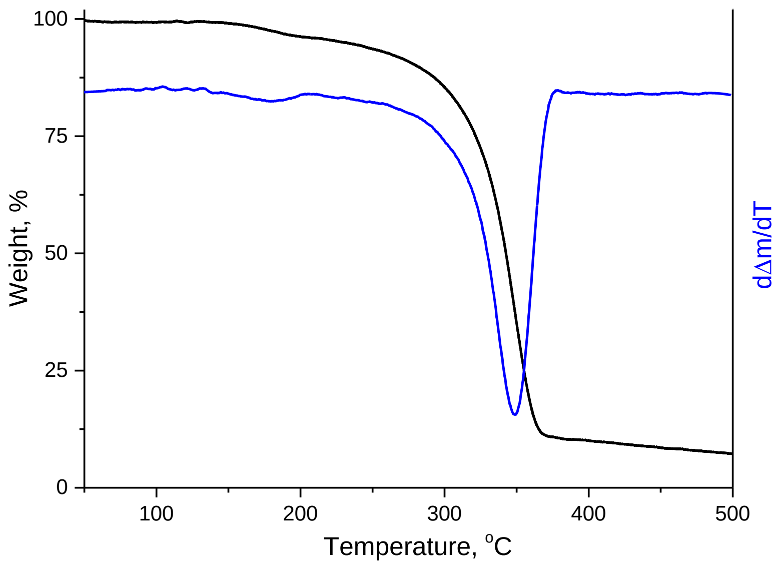

Figure S4. Dependency of weight loss (in %) and dΔm/dT on temperature for initial BC.
